# Association of Maternal Vitamin D Status with Glucose Tolerance and Caesarean Section in a Multi-Ethnic Asian Cohort: The Growing Up in Singapore Towards Healthy Outcomes Study

**DOI:** 10.1371/journal.pone.0142239

**Published:** 2015-11-16

**Authors:** See Ling Loy, Ngee Lek, Fabian Yap, Shu E. Soh, Natarajan Padmapriya, Kok Hian Tan, Arijit Biswas, George Seow Heong Yeo, Kenneth Kwek, Peter D. Gluckman, Keith M. Godfrey, Seang Mei Saw, Falk Müller-Riemenschneider, Yap-Seng Chong, Mary Foong-Fong Chong, Jerry Kok Yen Chan, Growing Up in Singapore Towards Healthy Outcomes (GUSTO) study group

**Affiliations:** 1 KK Research Centre, KK Women’s and Children’s Hospital, Singapore; 2 Duke-NUS Graduate Medical School, Singapore; 3 Department of Paediatrics, KK Women’s and Children’s Hospital, Singapore; 4 Singapore Institute for Clinical Sciences, Agency for Science, Technology and Research, (A*STAR), Singapore; 5 Department of Obstetrics and Gynaecology, Yong Loo Lin School of Medicine, National University of Singapore, National University Health Systems, Singapore; 6 Division of Obstetrics and Gynaecology, KK Women’s and Children’s Hospital, Singapore; 7 Department of Maternal Fetal Medicine, KK Women’s and Children’s Hospital, Singapore; 8 Liggins Institute, University of Auckland, Auckland, New Zealand; 9 MRC Lifecourse Epidemiology Unit, University of Southampton, Southampton, United Kingdom; 10 NIHR Southampton Biomedical Research Centre, University of Southampton and University Hospital Southampton NHS Foundation Trust, Southampton, United Kingdom; 11 Saw Swee Hock School of Public Health, National University of Singapore, Singapore; 12 Institute for Social Medicine, Epidemiology and Health Economics, Charite University Medical Centre, Berlin, Germany; 13 Department of Paediatrics, Yong Loo Lin School of Medicine, National University of Singapore and National University Health System, Singapore; 14 Clinical Nutrition Research Centre, Singapore Institute for Clinical Sciences (SICS), Agency for Science, Technology and Research (A*STAR), Singapore; 15 Department of Reproductive Medicine, KK Women’s and Children’s Hospital, Singapore; University of Alabama at Birmingham, UNITED STATES

## Abstract

**Objective:**

Epidemiological studies relating maternal 25-hydroxyvitamin D (25OHD) with gestational diabetes mellitus (GDM) and mode of delivery have shown controversial results. We examined if maternal 25OHD status was associated with plasma glucose concentrations, risks of GDM and caesarean section in the Growing Up in Singapore Towards healthy Outcomes (GUSTO) study.

**Methods:**

Plasma 25OHD concentrations, fasting glucose (FG) and 2-hour postprandial glucose (2HPPG) concentrations were measured in 940 women from a Singapore mother-offspring cohort study at 26–28 weeks’ gestation. 25OHD inadequacy and adequacy were defined based on concentrations of 25OHD ≤75nmol/l and >75nmol/l respectively. Mode of delivery was obtained from hospital records. Multiple linear regression was performed to examine the association between 25OHD status and glucose concentrations, while multiple logistic regression was performed to examine the association of 25OHD status with risks of GDM and caesarean section.

**Results:**

In total, 388 (41.3%) women had 25OHD inadequacy. Of these, 131 (33.8%), 155 (39.9%) and 102 (26.3%) were Chinese, Malay and Indian respectively. After adjustment for confounders, maternal 25OHD inadequacy was associated with higher FG concentrations (β = 0.08mmol/l, 95% Confidence Interval (CI) = 0.01, 0.14), but not 2HPPG concentrations and risk of GDM. A trend between 25OHD inadequacy and higher likelihood of emergency caesarean section (Odds Ratio (OR) = 1.39, 95% CI = 0.95, 2.05) was observed. On stratification by ethnicity, the association with higher FG concentrations was significant in Malay women (β = 0.19mmol/l, 95% CI = 0.04, 0.33), while risk of emergency caesarean section was greater in Chinese (OR = 1.90, 95% CI = 1.06, 3.43) and Indian women (OR = 2.41, 95% CI = 1.01, 5.73).

**Conclusions:**

25OHD inadequacy is prevalent in pregnant Singaporean women, particularly among the Malay and Indian women. This is associated with higher FG concentrations in Malay women, and increased risk of emergency caesarean section in Chinese and Indian women.

## Introduction

Vitamin D inadequacy, which is defined as serum 25-hydroxyvitamin D (25OHD) <75nmol/l in some studies [1–2] and <50nmol/l in others [2–3], is common among pregnant women and has become a global public health problem [4]. During pregnancy, the active form of vitamin D, 1,25-dihydroxyvitamin D (1,25(OH)_2_D) concentrations increase by 100% or more [5]. Recently, it has been shown that production of 20-hydroxyvitamin D (20OHD) was higher than 25OHD in placenta [6–7]. However, these hydroxyvitamin D metabolites are not routinely measured to reflect vitamin D pool of the body. Serum 25OHD which is the major circulating form of vitamin D, is currently used as the best determinant of vitamin D status [5]. Associations have been found between serum 25OHD with various pregnancy outcomes such as gestational diabetes mellitus (GDM), hyperglycaemia and caesarean section (S1 Table), but results have been inconclusive.

25OHD involves in glucose homeostasis via different mechanisms. In its active form, it improves insulin sensitivity of the target cells (liver, skeletal muscle and adipose tissue) [8]. It also improves *β*-cell function [9], protects *β*-cell from immune attacks and reduces insulin resistant through immunoregulatory and anti-inflammatory effects [8]. Serum 25OHD has been shown to be inversely associated with maternal fasting glucose [10–14] and postprandial glucose concentrations [15–16] during pregnancy, but there are conflicting data with respect to the risk of developing GDM. Some studies have suggested that low serum 25OHD was associated with GDM [12–13,16–18], while other studies found no significant associations with GDM [1,10,15,19–20].

It has been shown that both skeletal and uterine smooth muscle contain vitamin D receptors [21–22], with more recent data implicating 25OHD and regulation of contractile proteins in human uterine myometrial cells [23]. The relationship of 25OHD with labour and delivery outcomes can thus be related to both muscle performance and uterine contraction. Higher likelihood of caesarean delivery has been observed in pregnant women with low serum 25OHD concentrations [24–25], although this had not been the case in several other studies [1,19,26–27].

Serum 25OHD levels vary according to geographical location and sunlight exposure [28]. In fact, striking ethnicity disparities in the prevalence of 25OHD deficiency has been reported within the same country or even city [29–30]. The Third National Health and Nutrition Examination Survey (NHANES III) in United States found that serum 25OHD levels were inversely associated with diabetes risk in white, but not black populations [29]. However, an Australian study reported no association between serum 25OHD and GDM risk in any ethnic subgroups [10]. These studies between 25OHD, ethnicity and disease outcomes were mainly conducted in Western setting, but none have been designed to describe the association in a multi-ethnic Asian setting.

The Singapore Growing Up in Singapore Towards healthy Outcomes (GUSTO) study, consisting of mothers from three ethnic groups, namely the Chinese, Malays and Indians, provides a unique opportunity to evaluate pregnancy outcomes associated with 25OHD status across ethnic groups with the absence of seasonal variation in sunlight exposure. This study aimed to examine the association of maternal 25OHD status in the second trimester of pregnancy with plasma glucose concentrations, risks of GDM and caesarean section. We hypothesized that 25OHD inadequacy was associated with higher fasting glucose (FG) and 2-hour postprandial glucose (2HPPG) concentrations, increased risks of GDM and emergency caesarean section due to prolonged labour and foetal distress.

## Methods

### Study design and participants

Women were drawn from the GUSTO mother-offspring cohort study, which involved detailed assessment of pregnant women and characteristics of their offspring from birth onwards [31]. The GUSTO study was designed to investigate the effects of early life events on the risk of developing metabolic diseases in later life. This study was conducted according to the guidelines laid down in the Declaration of Helsinki. Ethical approval was obtained from the Domain Specific Review Board of Singapore National Healthcare Group (reference D/09/021) and the Centralised Institutional Review Board of SingHealth (reference 2009/280/D).

Pregnant women attending antenatal care (<14 weeks’ gestation) from June 2009 to September 2010 in KK Women’s and Children’s Hospital (KKH) and National University Hospital (NUH), which house the major public maternity units in Singapore, were recruited into the GUSTO study. The inclusion criteria included age range between 18 and 50 years, intention to reside in Singapore for the next five years, intention to deliver in KKH and NUH, and willingness to donate cord, cord blood and placenta. Only Chinese, Malay and Indian women whose parents and whose husband’s parents were of same ethnicity were included in the study. Women receiving chemotherapy, psychotropic drugs or with type 1 diabetes mellitus were excluded. Informed written consent was obtained from all women.

### Data collection

Women recruited in their first trimester returned to the hospitals at 26–28 weeks’ gestation for a follow-up visit. Detailed interviews were conducted in the clinics at recruitment and at 26–28 weeks’ gestation. Data on socioeconomic status, educational attainment, personal health, dietary supplement intake, smoking status and physical activity were collected. Smoking exposure was defined as current smoking or exposed to second hand smoke on a daily basis. After delivery, data on mode of delivery and complications were retrieved from the hospital case notes by trained health personnel.

### Physical activity assessment

Three types of physical activity were assessed, including light-moderate, moderate and vigorous intensity activities. Total level of physical activity was computed from the summation of the duration (in minutes) and frequency (days) of these three types of activity. Physical activity was expressed in metabolic equivalents (MET-minutes/week) and classified as not highly active (<3000 MET-minutes/week) and highly active (≥3000 MET minutes/week) levels [32].

### Anthropometric measurement

Maternal height was measured with a stadiometer (Seca 206, Hamburg, Germany). Maternal weight was based on body weight measured at first antenatal clinic visit during the first trimester of pregnancy. Body mass index (BMI) was computed from the formula: weight (kg)/ height (m^2^). Because obesity is a risk factor of low 25OHD [5], GDM and caesarean section [33], we therefore categorized the continuous values of BMI for analysis. The BMI was classified according to World Health Organization ranges: underweight <18.5kgm^-2^, normal weight 18.5–24.9kgm^-2^, overweight 25–29.9kgm^-2^ and obese ≥30.0kgm^-2^ [34].

### Plasma glucose and 25OHD concentrations

An overnight fasting blood samples were drawn at 26–28 weeks’ gestation for glucose and 25OHD analyses. At the same visit, women underwent 75g Oral Glucose Tolerance Test (OGTT) for GDM diagnosis using World Health Organization criteria (FG or 2HPPG concentrations ≥7.0 or ≥7.8mmol/l respectively) [35]. Plasma FG and 2HPPG concentrations were measured by colorimetry [Advia 2400 Chemistry system (Siemens Medical Solutions Diagnostics) and Beckman LX20 Pro analyzer (Beckman Coulter)].

Plasma 25OHD was analysed as 25-hydroxyvitamin D_2_ (25OHD_2_) and 25-hydroxyvitamin D_3_ (25OHD_3_) by isotope-dilution liquid chromatography–tandem mass spectrometry (ID-LC-MS/MS) [36]. The intra- and inter-assay CVs for 25OHD_2_ and 25OHD_3_ were ≤10.3%, and the detection limit was <4nmol/l for both metabolites. Women were categorized as having 25OHD inadequacy and 25OHD adequacy based on concentrations of 25OHD ≤75nmol/l and >75nmol/l respectively [37–38]. The cut-off of 25OHD deficiency at <50nmol/l was not adopted due to small sample size of pregnant women in this category when stratified by ethnicity, which would reduce the power of analysis (S2 Table).

### Statistical analysis

Categorical data were presented as frequencies and percentages, while continuous data were presented as means and standard deviations. Comparisons between maternal characteristics and 25OHD status were performed using Pearson’s Chi-square test for categorical variables and independent t-test for continuous variables. Multiple logistic regression analysis was used to assess the association of 25OHD status with risks of GDM and caesarean sections (total caesarean section, emergency caesarean section and related indications, and elective caesarean section). Multiple linear regression analysis was used to assess the association of 25OHD status with FG and 2HPPG concentrations.

Both logistic and linear regression models were adjusted for confounding variables, which included maternal age, parity, ethnicity, education, body mass index, smoking exposure, physical activity during pregnancy, and pre-existing diabetes and / or hypertension. These confounders were selected based on literature review [25,39–40]. Because neonatal sex has been reported to be associated with risk of maternal GDM [41–42] and since an association was found between 25OHD status and neonatal sex (p = 0.036), we therefore further adjusted for this confounding variable in the final model. All statistical analyses were performed using IBM SPSS statistics, Version 20 (USA). Two-sided tests were used. A value of *P<*0.05 was considered statistically significant.

## Results

**Fig 1:**
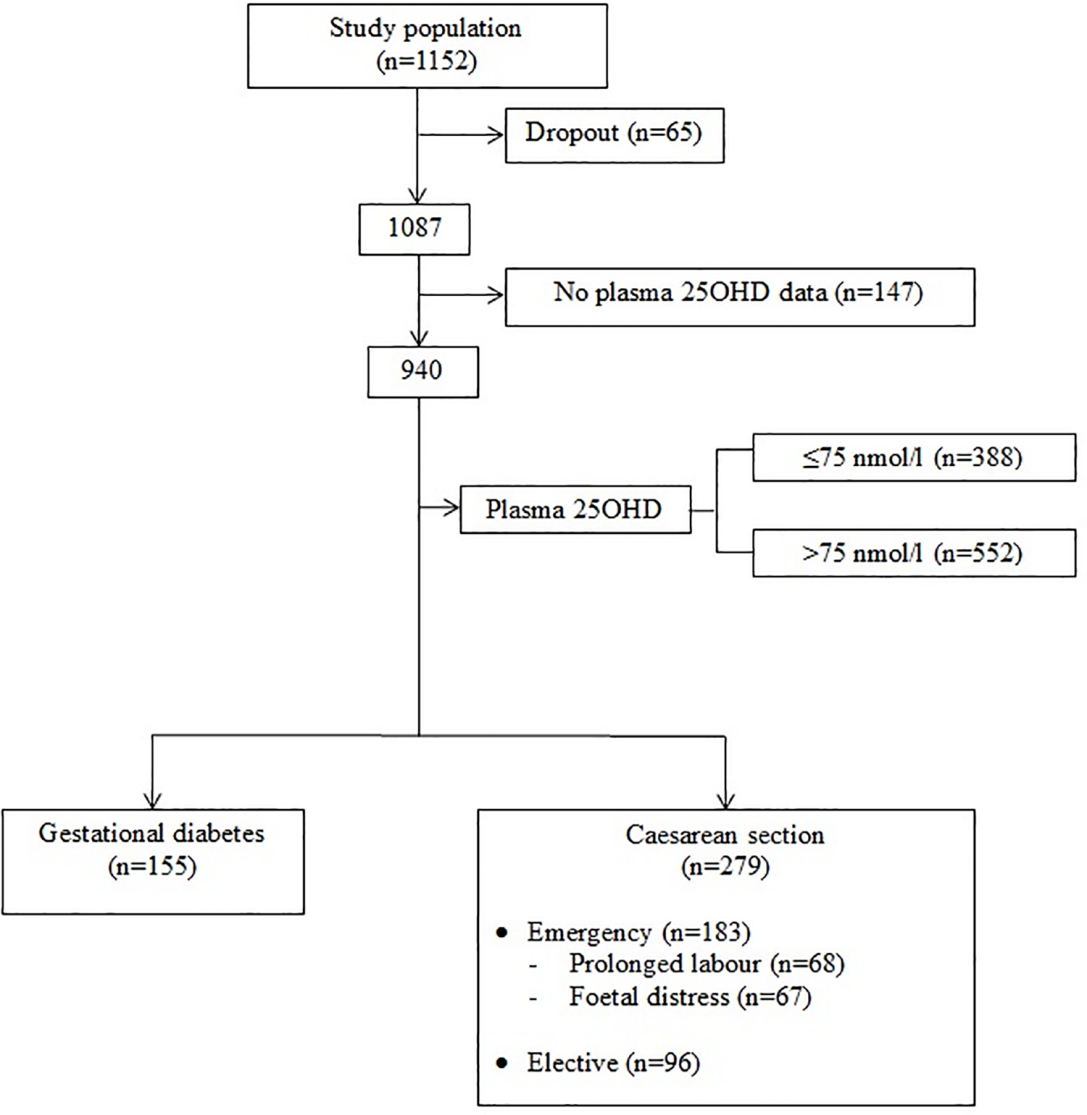
Study profile.

Of 1152 pregnant women who were recruited in the study at <14 weeks’ gestation, 1087 (94.4%) of them remained until the delivery stage (Fig 1). A total of 940 (86.5%) women with adequate amount of plasma samples were successfully analysed for 25OHD concentrations. Of this, 155 women had GDM, 279 women delivered via caesarean section, 183 and 96 women underwent emergency and elective caesarean sections respectively. Sixty eight women were delivered by emergency caesarean for a prolonged labour and another 67 for foetal distress. These two indications together accounted for almost three-quarters (73.8%) of delivery by emergency caesarean section (Table 1).

**Table 1:** Characteristics of participants according to 25OHD status (n = 940)^a^.

Characteristics	Total (n = 940)	25OHD inadequacy (n = 388)	25OHD adequacy (n = 552)	p^b^
Plasma 25OHD, nmol/l	81.03 (27.18)	55.43 (13.81)	99.02 (18.40)	<0.001
Age, years	30.53 (5.11)	29.54 (5.12)	31.24 (4.99)	<0.001
Ethnicity, n (%)				
Chinese	519 (55.2)	131 (25.2)	388 (74.8)	<0.001
Malay	247 (26.3)	155 (62.8)	92 (37.2)	
India	174 (18.5)	102 (58.6)	72 (41.4)	
Parity, n (%)				
Nulliparous	402 (42.8)	162 (40.3)	240 (59.7)	0.599
Multiparous	538 (57.2)	226 (42.0)	312 (58.0)	
Body mass index (kg/m^-2^)				
Underweight, n (%)	77 (8.2)	30 (39.0)	47 (61.0)	0.008
Normal weight, n (%)	571 (61.1)	222 (38.9)	349 (61.1)	
Overweight, n (%)	186 (19.9)	77 (41.4)	109 (58.6)	
Obese, n (%)	100 (10.7)	57 (57.0)	43 (43.0)	
Education, n (%)				
None/ Primary/ Secondary	284 (30.6)	117 (41.2)	167 (58.8)	0.007
Post-secondary	326 (35.2)	154 (47.2)	172 (52.8)	
University and others	317 (34.2)	111 (35.0)	206 (65.0)	
Smoking exposure, n (%)	344 (36.8)	163 (42.1)	181 (33.0)	0.004
Intake of supplement containing vitamin D and Calcium, n (%)	635 (74.6)	228 (68.1)	407 (78.9)	<0.001
Physical activity, n (%)				
Not highly active	746 (80.6)	308 (41.3)	438 (58.7)	0.881
Highly active	179 (19.4)	75 (41.9)	104 (58.1)	
Pre-existing diabetes and/ or hypertension, n (%)	19 (2.0)	8 (2.1)	11 (2.0)	0.941
Neonatal sex, n (%)				
Boys	489 (52.0)	186 (38.0)	303 (62.0)	0.036
Girls	451 (48.0)	202 (44.8)	249 (55.2)	
FG concentrations, mmol/l	4.35 (0.47)	4.39 (0.54)	4.31 (0.42)	0.020
2HPPG concentrations, mmol/l	6.49 (1.44)	6.43 (1.51)	6.53 (1.40)	0.303
GDM, n (%)	155 (17.7)	59 (16.8)	96 (18.3)	0.570
Caesarean section,^c^ n (%)	279 (29.7)	122 (31.4)	157 (28.4)	0.321
Emergency caesarean section, n (%)	183 (21.7)	86 (24.4)	97 (19.7)	0.101
Prolonged labour, n (%)	68 (8.1)	30 (8.5)	38 (7.7)	0.674
Foetal distress, n (%)	67 (7.9)	32 (9.1)	35 (7.1)	0.295
Elective caesarean section, n (%)	96 (10.2)	36 (11.9)	60 (13.2)	0.608

^a^ Total sample size is not always n = 940 due to the missing values. Data are presented as mean (standard deviation) or number (percentage). 25OHD inadequacy = 25OHD ≤75nmol/l; 25OHD adequacy = 25OHD>75nmol/l.

^b^ p values are determined by independent t-test or Pearson chi-square test.

^c^ Included both emergency and elective caesarean sections.

25OHD = 25-hydroxyvitamin D; FG = fasting glucose; 2HPPG = 2-hour postprandial glucose; GDM = gestational diabetes mellitus

Table 1 shows the characteristics of the participants. The mean 25OHD concentration for all women (n = 940) was 81.0nmol/l (standard deviation = 27.2). A total of 388 (41.3%) women had 25OHD inadequacy. Compared to women with adequate 25OHD status, this group of women were younger (p<0.001), comprised of more Malays (p<0.001), heavier (p = 0.008), attained lower educational levels (p = 0.007), reported higher smoking exposures during pregnancy (p = 0.004) and less likely to take vitamin D and calcium supplements during pregnancy (p<0.001). No significant differences in maternal 25OHD status were observed when analysed for parity, physical activity and pre-existing diabetes and/ or hypertension. Higher FG concentrations were found in women with 25OHD inadequacy compared to those with 25OHD adequacy (p = 0.020). Upon stratification by ethnicity, FG concentrations and incidence of emergency caesarean sections differed by 25OHD status in only the Malay (p = 0.027) and Indian women (p = 0.034) (Table 2).

**Table 2:** Pregnancy outcomes according to 25OHD status and ethnicity.

Variables	Chinese (n = 519)			Malay (n = 247)			Indian (n = 174)		
	25OHD inadequacy (n = 131)	25OHD adequacy (n = 388)	p^a^	25OHD inadequacy (n = 155)	25OHD adequacy (n = 92)	p^a^	25OHD inadequacy (n = 102)	25OHD adequacy (n = 72)	p^a^
FG concentrations, mmol/l	4.33 (0.51)	4.31 (0.42)	0.741	4.39 (0.60)	4.25 (0.38)	**0.027**	4.48 (0.49)	4.39 (0.46)	0.266
2HPPG concentrations, mmol/l	6.67 (1.43)	6.60 (1.40)	0.622	6.20 (1.49)	6.20 (1.31)	0.995	6.47 (1.62)	6.59 (1.46)	0.629
GDM	24 (20.0)	75 (20.3)	0.949	14 (9.8)	8 (9.0)	0.839	21 (23.6)	13 (19.4)	0.530
Caesarean section^b^	42 (32.1)	104 (26.8)	0.247	40 (25.8)	30 (32.6)	0.251	40 (39.2)	23 (31.9)	0.326
Emergency caesarean section	27 (23.3)	65 (18.6)	0.276	27 (19.0)	21 (25.3)	0.267	32 (34.0)	11 (18.3)	**0.034**
Prolonged labour	10 (8.6)	27 (7.7)	0.760	13 (9.2)	8 (9.6)	0.904	7 (7.4)	3 (5.0)	0.548
Foetal distress	8 (6.9)	21 (6.0)	0.734	14 (9.9)	9 (10.8)	0.814	10 (10.6)	5 (8.3)	0.638
Elective caesarean section	15 (14.4)	39 (12.1)	0.531	13 (10.2)	9 (12.7)	0.587	8 (11.4)	12 (19.7)	0.191

Data are presented as mean (standard deviation) or number (percentage). 25OHD inadequacy = 25OHD ≤75nmol/l; 25OHD adequacy = 25OHD>75nmol/l. The values in bold indicate p<0.05.

^a^ p values are determined by chi-square test or independent t-test.

^b^ Included both emergency and elective caesarean sections.

25OHD = 25-hydroxyvitamin D; FG = fasting glucose; 2HPPG = 2-hour postprandial glucose; GDM = gestational diabetes mellitus.

Table 3 presents the associations between 25OHD status and related outcomes before and after adjusting for potential confounding. Compared to women with adequate 25OHD status, women with 25OHD inadequacy had higher FG concentrations (β = 0.08mmol/l, 95% Confidence Interval (CI) = 0.02, 0.14). This effect estimate did not change even after adjustment for confounders (β = 0.08mmol/l, 95% CI = 0.01, 0.14). Women with 25OHD inadequacy showed a trend towards a higher likelihood of emergency caesarean section in both unadjusted (Odds Ratio (OR) = 1.32, 95% CI = 0.95, 1.83) and adjusted (OR = 1.39, 95% CI = 0.95, 2.05) models. The 2HPPG concentrations, GDM and non-emergency caesarean section rates were not found to be associated with 25OHD status in both unadjusted and adjusted models.

**Table 3:** Associations between 25OHD status and pregnancy outcomes.

	Crude			Adjusted		
Maternal outcomes	25OHD adequacy	25OHD inadequacy		25OHD adequacy	25OHD inadequacy	
		OR (95% CI)^a^	p		OR (95% CI)^a^	p
FG concentrations, mmol/l	reference	0.08 (0.02, 0.14)^b^	**0.014**	Reference	0.08 (0.01, 0.14)^b^	**0.025**
2HPPG concentrations, mmol/l	reference	-0.10 (-0.30, 0.09)^b^	0.303	Reference	0.05 (-0.15, 0.25)^b^	0.631
GDM	reference	0.90 (0.63, 1.29)	0.571	Reference	1.02 (0.68, 1.53)	0.938
Caesarean section^c^	reference	1.15 (0.87, 1.53)	0.322	Reference	1.15 (0.83, 1.58)	0.406
Emergency caesarean section	reference	1.32 (0.95, 1.83)	0.102	Reference	1.39 (0.95, 2.05)	0.092
Prolonged labour	reference	1.11 (0.68, 1.83)	0.674	Reference	1.24 (0.68, 2.27)	0.480
Foetal distress	reference	1.31 (0.79, 2.15)	0.296	Reference	1.08 (0.61, 1.91)	0.788
Elective caesarean section	reference	0.89 (0.57, 1.39)	0.608	Reference	0.76 (0.47, 1.24)	0.277

Adjusted for maternal age, parity, ethnicity, education, body mass index, smoking exposure, physical activity, pre-existing diabetes and/ or hypertension, neonatal sex. 25OHD inadequacy = 25OHD ≤75nmol/l; 25OHD adequacy = 25OHD>75nmol/l. The values in bold indicate p<0.05.

^a^ Data are presented as Odds Ratio (95% Confidence Interval), unless otherwise indicated.

^b^ Data are presented as β regression coefficient (95% Confidence Interval)

^c^ Included both emergency and elective caesarean sections.

25OHD = 25-hydroxyvitamin D; FG = fasting glucose; 2HPPG = 2-hour postprandial glucose; GDM = gestational diabetes mellitus

When analyses were stratified by ethnicity (Table 4), the association between inadequate 25OHD status and higher FG concentrations was significant in Malay women (β = 0.19 mmol/l, 95% CI = 0.04, 0.33), but not in Chinese and Indian women, while the odds of having emergency caesarean section were approximately two times greater in Chinese (OR = 1.90, 95% CI = 1.06, 3.43) and Indian women (OR = 2.41, 95% CI = 1.01, 5.73) with 25OHD inadequacy compared to those with 25OHD adequacy.

**Table 4:** Associations between 25OHD status and pregnancy outcomes according to ethnicity.

	Chinese			Malay			Indian		
Pregnancy outcomes	25OHD adequacy	25OHD inadequacy		25OHD adequacy	25OHD inadequacy		25OHD adequacy	25OHD inadequacy	
		OR^a^ (95% CI)	P		OR^a^ (95% CI)	p		OR^a^ (95% CI)	p
FG concentrations, mmol/l	reference	0.03 (-0.06, 0.12)^b^	0.507	Reference	0.19 (0.04, 0.33)^b^	**0.013**	reference	0.09 (-0.07, 0.24)^b^	0.268
2HPPG concentrations, mmol/l	reference	0.10 (-0.21, 0.36)^b^	0.616	Reference	0.12 (-0.24, 0.49)^b^	0.509	reference	0.08 (-0.42, 0.59)^b^	0.747
GDM	reference	0.92 (0.54, 1.59)	0.771	Reference	1.36 (0.50, 3.72)	0.546	reference	01.34 (0.55, 3.24)	0.520
Caesarean section^c^	reference	1.46 (0.91, 2.35)	0.117	Reference	0.67 (0.37, 1.23)	0.194	reference	1.42 (0.70, 2.89)	0.337
Emergency caesarean section	reference	1.90 (1.06, 3.43)	**0.033**	Reference	0.56 (0.28, 1.15)	0.115	reference	2.41 (1.01, 5.73)	**0.048**
Prolonged labour	reference	1.91 (0.80, 4.58)	0.146	Reference	0.76 (0.27, 2.14)	0.599	reference	1.50 (0.32, 7.04)	0.608
Foetal distress	reference	1.41 (0.55, 3.59)	0.477	Reference	0.73 (0.28, 1.94)	0.533	reference	1.33 (0.33, 5.29)	0.690
Elective caesarean section	reference	1.02 (0.52, 2.01)	0.948	Reference	0.98 (0.37, 2.61)	0.967	reference	0.35 (0.12, 1.01)	0.052

Adjusted for maternal age, parity, education, body mass index, smoking exposure, physical activity, pre-existing diabetes and/ or hypertension, neonatal sex. 25OHD inadequacy = 25OHD ≤75nmol/l; 25OHD adequacy = 25OHD>75nmol/l. The values in bold indicate p<0.05.

^a^ Data are presented as Odds Ratio (95% Confidence Interval), unless otherwise indicated.

^b^ Data are presented as β regression coefficient (95% Confidence Interval)

^c^ Included both emergency and elective caesarean sections.25OHD = 25-hydroxyvitamin D; FG = fasting glucose; 2HPPG = 2-hour postprandial glucose; GDM = gestational diabetes mellitus.

## Discussion

In this multi-ethnic cohort, 41% of pregnant women were found to have inadequate plasma levels of 25OHD in the second trimester, with substantially higher rates found in Malay and Indian women compared to Chinese women. Overall, maternal 25OHD inadequacy was significantly associated with higher FG concentrations, and a trend towards higher likelihood of emergency caesarean delivery. By ethnicity, the association between inadequate 25OHD status and higher FG concentrations was found to be significant only in Malay women, while the odds of having emergency caesarean section were approximately two times greater in Chinese and Indian women with 25OHD inadequacy. We found no association of maternal 25OHD status with 2HPPG concentrations, risks of GDM and non-emergency caesarean sections in the overall cohort as well as within any ethnic group.

Our observation mostly supports the findings of previous studies regarding the association between 25OHD and glucose metabolism in obstetric populations. In line with previous studies [10–14], we found an inverse association between 25OHD and FG concentrations, but no association with 2HPPG concentrations was observed [11,13]. This suggests that 2HPPG may be less likely to be influenced by 25OHD concentrations although its variability is larger than FG. The reasons for the lack of an association between 25OHD and 2HPPG are unclear. It has been reported that 1- and 2-hour postprandial glucose values are largely driven by insulin resistant state [11]. One possibility is that 25OHD may not influence glucose metabolism via modulation of insulin sensitivity, but through other pathways such as modulating pancreatic beta-cell function or cytokines generation [43]. Compared to 2HPPG, FG during pregnancy has been shown as a stronger predictor of foetal serum C-peptide levels [44] and adiposity [45], suggesting a potential link between maternal 25OHD inadequacy and impaired metabolic outcomes in the offspring.

While two previous studies from Australia involving approximately 300 women have shown an inverse association between 25OHD and FG independent of ethnicity [10–11], we found that similar to the NHANES III among non-pregnant population [29], ethnicity can modify the association between 25OHD and glucose metabolism. When ethnic subgroups were analysed separately, the inverse association between 25OHD and FG only appeared significant in Malay women, but not in Chinese and Indian women, suggesting the importance of vitamin D adequacy to optimise FG concentrations in Malay pregnant women. The inverse association between 25OHD and FG in Malay women could also be attributable to their relatively higher rates of 25OHD deficiency (25OHD<50nmol/l) compared to other ethnic groups. However, the contrasting lack of any inverse association of 25OHD with FG in Indian women was unexpected given their relatively lower 25OHD concentrations and higher FG concentrations. It is possible that this group of Indian women were predisposed to the risk of fasting hyperglycaemia through pathway which is independent from the influence of 25OHD concentrations [10].

The association between serum 25OHD and GDM in the literature is conflicting [46]. In general, the association between 25OHD and GDM was more commonly shown in populations with high prevalence of 25OHD deficiency. In a Turkish study, Zuhur and colleagues [13] showed significantly lower 25OHD levels in GDM women (n = 234, mean = 30.8nmol/l) than the controls (n = 168, mean = 36.0nmol/l). However, when subgroups of 25OHD levels were analysed, only women with severely deficient 25OHD (n = 64, <12.5nmol/l) showed an increased risk of GDM [13]. Similarly, in a Chinese study of 400 pregnant women with 54% 25OHD deficiency (<25nmol/l), a significant association between 25OHD at 26–28 weeks’ gestation and risk of developing GDM was found [12]. Similar observations were shown by other studies which reported a high prevalence (>25%) of vitamin D deficiency with 25OHD<50nmol/l [16–18]. In our cohort, only 0.5% of women had 25OHD<25nmol/l and 13.4% had 25OHD<50nmol/l, and this may explain the lack of association between 25OHD status and GDM. In fact, several other studies performed in populations where 25OHD deficiency is low [15,20] shared the same finding as ours. We recognized that some of the discrepancies in findings between 25OHD and GDM could also be due to the use of different GDM diagnosis criteria across studies.

In a cross-sectional study of 253 women, 25OHD<37.5nmol/l measured at birth was associated with a fourfold increased risk of caesarean section [24]. This finding was replicated by Scholl et al. [25] who reported a less than twofold (66%) increased risk of caesarean birth for women with 25OHD<30nmol/l measured at 14 weeks’ gestation. When specific indications were examined, 25OHD<30nmol/l was linked to a twofold increased risk of caesarean for prolonged labour [25]. While our data did not show any significant association between 25OHD status and total caesarean section, we found that Chinese and Indian women with 25OHD inadequacy were approximately two times more likely to have emergency caesarean section than those with 25OHD adequacy. A prospective cohort which studied 995 women in UK reported no association of 25OHD in the first trimester with emergency or elective caesarean sections, and related indications such as failure to progress and foetal distress in labour [26]. Similarly, we also did not observe any significant association of 25OHD status with risks of prolonged labour and foetal distress which could be due to small sample size. These null findings were supported by others [1,19,27]. In these studies, 25OHD assessment was done in early pregnancy [19,26] and the effect on mode of delivery may become apparent only in late pregnancy [26].

The strengths of this study include its prospective study design [31] and the use of liquid chromatography–tandem mass spectrometry which is a more accurate method compared to other techniques in measuring 25OHD concentrations [47]. Our study was limited for not being able to adjust for residual confounding. Only one blood sample during mid-late gestation was obtained which restricts our ability to evaluate 25OHD concentrations in other windows that could have had a profound impact on maternal plasma glucose, GDM risk or mode of delivery. Other pregnancy outcomes such as preeclampsia or infections were not examined due to their relatively low prevalence rates in our study. A growing body of literature has now documented an association of genetic variation in cytochrome P450, vitamin D binding protein [48] and vitamin D receptor [49] with 25OHD concentrations that could impact on vitamin D metabolism and disease susceptibility. These genetic factors might shed some light in the ethnicity susceptibility in the association between 25OHD and different outcome measures. Thus, assessment of vitamin D related genotype and stratification of cases by both serum levels and genetic polymorphisms are warranted in future studies.

## Conclusions

In conclusion, while prevalence of 25OHD deficiency is low, 25OHD inadequacy is highly prevalent during pregnancy in Singaporean women, particularly among Malay and Indian women. This is associated with higher FG concentrations in Malay women and an increased risk of emergency caesarean section in Chinese and Indian women. This may suggest varying threshold effects of 25OHD sensitivity on pregnancy outcomes among ethnic groups. Further investigations on biological components, social, nutritional practices and cultural differences are required to explain the mechanism of ethnicity disparity in 25OHD effects. Nevertheless, the present findings are important to provide evidence for clinical recommendations regarding potential screening of 25OHD inadequacy during prenatal care and the need for vitamin D supplementation in at risk groups.

## Supporting Information

S1 Table Studies on maternal 25OHD status and pregnancy outcomes. (DOCX)

S2 Table Characteristics of pregnant women (n = 940). (DOC)
