# Supplementary material for: Association of Maternal Vitamin D Status with Glucose Tolerance and Caesarean Section in a Multi-Ethnic Asian Cohort: The Growing Up in Singapore Towards Healthy Outcomes Study
Source: PLoS One. 2015 Nov 16;10(11):e0142239. doi: 10.1371/journal.pone.0142239 (PMC4646602; doi:10.1371/journal.pone.0142239)
Supplement: S1 Table — (DOCX) [file pone.0142239.s001.docx]

# Supporting Information

**S1 Table. Studies on maternal 25OHD status and pregnancy outcomes**

| Study | Country | Study design | Sample size (n) | Specimen GA  (weeks) | 25OHD cut-off  (nmol/l) | Outcomes | Statistics |
| --- | --- | --- | --- | --- | --- | --- | --- |
| Clifton-Bligh et al. 2008 | Australia | Prospective cohort | 265 | >96% in 2^nd^ – 3^rd^ trimester | <50 vs. ≥50 | GDM | AOR (95% CI): 1.92 (0.89, 4.17) |
|  |  |  | 264 |  | ln (25OHD) | Fasting glucose | ABC (95% CI): -0.13 (-0.26, 0.01) |
| McLeod et al. 2011 | Australia | Cross-sectional | 399 | 24-32 | Per 1 SD increase | Fasting glucose | ABC (95% CI): -0.05 (-0.08, -0.01) |
|  |  |  |  |  | 25OHD (continuous) | 1HPPG | Pearson’s correlation coefficient: 0.002, p=0.973 |
|  |  |  |  |  | 25OHD (continuous) | 2HPPG | Pearson’s correlation coefficient: 0.078, p=0.122 |
| Wang et al. 2012 | China | Case-control | 400 | 26-28 | <25 vs. ≥25 | GDM | AOR (95% CI): 1.59 (1.03, 2.44) |
|  |  |  |  |  | 25OHD (continuous) | Fasting glucose (control group) | Pearson’s correlation coefficient: -0.18, p=0.009 |
| Zuhur et al. 2013 | Turkey | Cross-sectional | 402 | 24-28 | <12.5 vs. ≥12.5 | GDM | AOR (95% CI): 3.95 (1.68, 9.25) |
|  |  |  |  |  | 25OHD (continuous) | Fasting glucose | Pearson’s correlation coefficient: -0.18, p<0.0001 |
|  |  |  |  |  | 25OHD (continuous) | 2HPPG | Pearson’s correlation coefficient: -0.09, p=0.10 |
| El Lithy et al., 2014 | Egypt | Case-control | 160 | 3^rd^ trimester | 25OHD (continuous) | Fasting glucose | Pearson’s correlation coefficient: -0.245, p<0.001 |
| Burris et al. 2012 | USA | Prospective cohort | 1314 | 26-28 | <25 vs. ≥25 | GDM | AOR (95% CI): 2.20 (0.80, 5.50) |
|  |  |  |  |  |  | 1HPPG | ABC (95% CI): 7.20 (0.20, 14.2) |
| Parlea et al. 2011 | Canada | Case-control | 335 | 15-18 | <73.5 vs. ≥73.5 | GDM | AOR (95% CI): 2.21 (1.19, 4.13) |
|  |  |  |  |  | 25OHD (continuous) | 1HPPG | Spearman’s rank correlation coefficient: -0.18, p<0.001 |
| Cho et al. 2013 | Korea | Case-control | 60 | 38 (mean) | <50 vs. ≥50 | GDM | AOR (95% CI): 30.78 (4.65, 203.90) |
| Lacroix et al. 2014 | Canada | Prospective cohort | 655 | 6-13 | Per 1 SD decrease | GDM | AOR (95% CI): 1.48 (1.03, 2.12) |
| Schneuer et al. 2014 | Australia | Case control | 5109 | 10-14 | <25 vs. 50-75 | GDM | AOR (95% CI): 0.97 (0.56, 1.69) |
| Charatcharoenwitthaya et al. 2013 | Thailand | Prospective cohort | 120 | 1^st^ and 2^nd^ trimesters | <75 vs. ≥75 | GDM | χ^2^ test/ Fisher exact test (1^st^ trimester), p=0.42  χ^2^ test/ Fisher exact test (2^nd^ trimester), p=0.91 |
|  |  |  |  | 1^st^ and 3^rd^ trimesters | <75 vs. ≥75 | Caesarean section | OR (95% CI) (1^st^ trimester): 2.09 (0.63, 6.91)  OR (95% CI) (3^rd^ trimester): 1.77 (0.72, 4.83) |
| Fernández-Alonso et al. 2012 | Spain | Cross-sectional | 466 | 11-14 | <50 vs. ≥75 | GDM | χ^2^ test, p=0.32 |
|  |  |  |  |  |  | Caesarean section | χ^2^ test p=0.65 |
|  |  |  |  |  |  | Emergency caesarean section | χ^2^ test, p=0.47 |
|  |  |  |  |  |  | Elective caesarean section | χ^2^ test, p=0.06 |
| Merewood et al. 2009 | USA | Cross-sectional | 253 | At birth | <37.5 vs. ≥37.5 | Primary caesarean section | AOR (95% CI): 3.84 (1.71, 8.62) |
| Scholl et al. 2012 | USA | Prospective cohort | 1153 | 13.73 (mean) | <30 vs. 50-125 | Caesarean section | AOR (95% CI): 1.66 (1.09, 2.52) |
|  |  |  |  |  |  | Prolonged labour | AOR (95% CI): 2.08 (1.09, 3.98) |
|  |  |  |  |  |  | Foetal distress | AOR (95% CI): 1.54 (0.64, 3.70) |
| Savvidou et al. 2012 | UK | Prospective cohort | 995 | 11-13 | 25OHD (continuous) | Emergency caesarean section | Mann-Whitney U test, p=0.53 |
|  |  |  |  |  | 25OHD (continuous) | Failure to progress in labour | Mann-Whitney U test, p=0.25 |
|  |  |  |  |  | 25OHD (continuous) | Foetal distress in labour | Mann-Whitney U test, p=0.72 |
|  |  |  |  |  | 25OHD (continuous) | Elective caesarean section | Mann-Whitney U test, p=0.81 |
| Gernand et al. 2014 | USA | Prospective cohort | 2798 | ≤26 | <30 vs. ≥75 | Primary caesarean section | ARR (95% CI): 0.65 (0.24, 1.81) |
|  |  |  |  |  |  | Prolonged labour stage 1 | ARR (95% CI): 1.37 (0.75, 2.53) |
|  |  |  |  |  |  | Prolonged labour stage 2 | ARR (95% CI): 1.59 (0.74, 3.42) |

25OHD, 25-hydroxyvitamin D; GA, gestational age at the time of specimen sampling; SD, standard deviation; GDM, gestational diabetes mellitus; 1HPPG, 1-hour postprandial glucose; 2HPPG, 2-hour postprandial glucose; AOR, adjusted odds ratio; CI, confidence interval; ABC, adjusted beta coefficient; ARR, adjusted risk ratio
