# Supplementary material for: Association of Maternal Vitamin D Status with Glucose Tolerance and Caesarean Section in a Multi-Ethnic Asian Cohort: The Growing Up in Singapore Towards Healthy Outcomes Study
Source: PLoS One. 2015 Nov 16;10(11):e0142239. doi: 10.1371/journal.pone.0142239 (PMC4646602; doi:10.1371/journal.pone.0142239)
Supplement: S2 Table — (DOC) [file pone.0142239.s002.doc]

# Supporting Information

| **S2 Table. Characteristics of pregnant women (n=940)^a^.** | | | | |
| --- | --- | --- | --- | --- |
| Characteristics | 25OHD <50nmol/l  (n=126) | 25OHD 50-75nmol/l  (n=262) | 25OHD >75nmol/l  (n=552) | p^b^ |
| Plasma 25OHD, nmol/l | 38.71 (7.49) | 63.46 (7.51) | 99.02 (18.40) | <0.001 |
| Age, years | 28.97 (5.28) | 29.81 (5.03) | 31.24 (4.99) | <0.001 |
| Ethnicity, n (%) |  |  |  |  |
| Chinese | 27 (5.2) | 104 (20.0) | 388 (74.8) | <0.001 |
| Malay | 60 (24.3) | 95 (38.5) | 92 (37.2) |  |
| India | 39 (22.4) | 63 (36.2) | 72 (41.4) |  |
| Parity, n (%) |  |  |  |  |
| Nulliparous | 47 (11.7) | 115 (28.6) | 240 (59.7) | 0.409 |
| Multiparous | 115 (28.6) | 147 (27.3) | 312 (58.0) |  |
| Body mass index, kgm^-2^ |  |  |  |  |
| Underweight, n (%) | 9 (11.7) | 21 (27.3) | 47 (61.0) | 0.061 |
| Normal weight, n (%) | 72 (12.6) | 150 (26.3) | 349 (61.1) |  |
| Overweight, n (%) | 25 (13.4) | 52 (28.0) | 109 (58.6) |  |
| Obese, n (%) | 20 (20.0) | 37 (37.0) | 43 (43.0) |  |
| Education, n (%) |  |  |  |  |
| None/ Primary/ Secondary | 47 (16.5) | 70 (24.6) | 167 (58.8) | 0.004 |
| Post-secondary | 42 (12.9) | 112 (34.4) | 172 (52.8) |  |
| University and others | 34 (10.7) | 77 (24.3) | 206 (65.0) |  |
| Smoking exposure, n (%) | 64 (51.2) | 99 (37.8) | 181 (33.0) | 0.001 |
| Intake of supplement containing vitamin D and Calcium, n (%) | 62 (60.8) | 166 (71.2) | 407 (78.9) | <0.001 |
| Physical activity, n (%) |  |  |  |  |
| Not highly active | 101 (13.5) | 207 (27.7) | 438 (58.7) | 0.980 |
| Highly active | 24 (13.4) | 51 (28.5) | 104 (58.1) |  |
| Pre-existing diabetes and/ or hypertension, n (%) | 0 | 8 (3.1) | 11 (2.0) | 0.135 |
| GDM, n (%) | 16 (14.2) | 43 (18.0) | 96 (18.3) | 0.578 |
| FG concentrations, mmol/l | 4.42 (0.57) | 4.38 (0.53) | 4.31 (0.42) | 0.035 |
| 2HPPG concentrations, mmol/l | 6.17 (1.32) | 6.55 (1.59) | 6.53 (1.40) | 0.042 |
| Caesarean section,^c^n (%) | 39 (31.0) | 83 (31.7) | 157 (28.4) | 0.605 |
| Emergency caesarean section, n (%) | 28 (24.3) | 58 (24.5) | 97 (19.7) | 0.261 |
| Prolonged labour, n (%) | 11 (9.6) | 19 (8.0) | 38 (7.7) | 0.808 |
| Foetal distress, n (%) | 11 (9.6) | 21 (8.9) | 35 (7.1) | 0.563 |
| Elective caesarean section, n (%) | 11 (11.2) | 25 (12.3) | 60 (13.2) | 0.849 |
| ^a^ Total sample size is not always n=940 due to the missing values. Data are presented as mean (standard deviation) or number (percentage).  ^b^p values are determined by ANOVA test or Pearson chi-square test.  ^c^ Included both emergency and elective caesarean sections.  25OHD=25-hydroxyvitamin D; FG=fasting glucose; 2HPPG=2-hour postprandial glucose; GDM=gestational diabetes mellitus | | | | |
